# Genome Sequences of Microbacterium foliorum Bacteriophages StrawberryJamm, Quammi, Teehee, and Casend

**DOI:** 10.1128/mra.00185-22

**Published:** 2022-04-06

**Authors:** Thomas P. Hoag, Joshua P. DeKlotz, Brian Steele, William F. Ettinger, Kristen A. Butela, Kirk R. Anders, Marianne Poxleitner

**Affiliations:** a Department of Biology, Gonzaga University, Spokane, Washington, USA; b Department of Biological Sciences, University of Pittsburgh, Pittsburgh, Pennsylvania, USA; DOE Joint Genome Institute

## Abstract

Four microbacteriophages infecting the host Microbacterium foliorum were isolated at Gonzaga University as part of the SEA-PHAGES program. Phages Teehee, StrawberryJamm, Quammi, and Casend are in the EG cluster, with average genome sizes of 62,263 bp and GC contents of 67.2%, with other interesting characteristics.

## ANNOUNCEMENT

Studying the vast diversity of bacteriophages provides information about the genetic structures and functions of bacteriophages. Four phages, Teehee, StrawberryJamm, Quammi, and Casend, were isolated from dry soil samples on Gonzaga University’s campus in Spokane, WA, using bacterial host Microbacterium foliorum NRRL B-24224, and their genomes were sequenced. Enriched isolations were performed at 25°C by shaking soil samples in PyCa media for 1 week in the presence of *M. foliorum*, followed by three rounds of plaque purification. DNA was isolated from lysates using Promega DNA Wizard kits. Each genome was sequenced using the Illumina sequencing platform at the Pittsburgh Bacteriophage Institute. Libraries were prepared from genomic DNA using NEB Ultra II FS DNA kits and an Illumina MiSeq instrument, yielding at least 700,000 150-base paired-end reads per sample. Raw reads were assembled using Newbler v2.6 (1) and Consed v29 (2) into a single contig for each phage. All four phages have a genome size of just over 60,000 bp, with direct terminal repeats of approximately 200 bp, all of which are characteristic of the EG cluster (Table 1).

**TABLE 1:** Display of genome size, fold coverage, GC content, length of terminal repeat, putative functions, and closest relative with the percent identity using NCBI BLASTn for the four phages (with GPS coordinates) Casend (47.671731, −117.402676), Quammi (47.667333, −117.402917), StrawberryJamm (47.667490, −117.404954), and Teehee (47.666208, −117.406311)^*a*^

Phage	Genome size (bp)	Fold coverage	GC content (%)	Terminal repeat (bp)	Putative functions/total no. of genes	Closest relative and % identity
Casend	62,461	2,270	66.9	204	32/106	Teehee, 99.8
Quammi	61,903	1,475	66.9	203	37/107	Rudy, 99.7
StrawberryJamm	61,953	224	67.0	198	37/113	Quammi, 97.6
Teehee	62,291	549	67.0	199	29/106	Casend, 99.8

a Casend contains 106 protein-coding genes, Quammi contains 107 protein-coding genes, StrawberryJamm contains 113 protein-coding genes, and Teehee contains 106 protein-coding genes. Putative functions represent the number of genes within each phage that were assigned a known function. Rudy is available at GenBank with accession no. MW365946.

Genomes were manually annotated using Glimmer v3.02 (3), GeneMarkS v2.5p (4), DNA Master 5.0.2 (http://cobamide2.bio.pitt.edu/computer.htm), PhagesDB BLAST, NCBI BLAST v2.10 (5), HHPRED (4), Conserved Domain Database, tRNA Scan SE v2.0 (6), ARAGORN v1.2 (7), TmHmm v2.0 (8), and PECAAN according to the SEA-PHAGES bioinformatics guide (9). No tRNA sequences were identified for Teehee, Casend, or Quammi. StrawberryJamm has one tRNA^Ser^ at bp 42449 to 42360. Putative functions were assigned to genes in all four phages (Table 1). NCBI BLASTn was used to search for similarity and identity against other published genomes (Table 1). The results of BLASTn against PhagesDB, which consists of published and nonpublished genomes, were consistent with the NCBI BLASTn data.

At the time of phage annotation, StrawberryJamm had four orphan genes (*3*, *56*, *89*, and *105*), Casend had two (*1* and *2*), and Quammi had two (*67* and *90*). Teehee had no orphan genes. The four phages each contain only one endolysin gene, display clear lytic plaque morphology, and lack repressor and integrase genes necessary for lysogeny, suggesting that the phages are lytic (10). Genes coding for holin and scaffolding protein are highly conserved in other phage clusters but are not found in any of the annotated EG phages, including these four phages. Following the gene coding for endolysin, the second halves of the four genomes are transcribed in the reverse direction and contain genes associated with membrane proteins. Each phage has multiple putative membrane proteins identified by TmHmm v2.0 and SOSUI. There are several putative protein products associated with DNA binding functions such as single-stranded DNA (ssDNA) binding protein and DNA primase, which are highly conserved among other EG phages. StrawberryJamm and Teehee also lack a histidine triad nucleotide binding protein gene which is present in both Quammi and Casend. Otherwise, the four EG phages are similarly organized, beginning with a section of variable small genes transcribed in the reverse direction.

### Data availability.

Teehee is available at GenBank with accession no. MW712727.1. Sequencing reads are part of the Sequence Read Archive with SRR18112518 under BioProject PRJNA488469. StrawberryJamm is available at GenBank with accession no. MW712724.1. Sequencing reads are part of the Sequence Read Archive with SRR18112519 under BioProject PRJNA488469. Quammi is available at GenBank with accession no. MW712728.1. Sequencing reads are part of the Sequence Read Archive with SRR18112520 under BioProject PRJNA488469 Casend is available at GenBank with accession no. MT657333.1. Sequencing reads are part of the Sequence Read Archive with SRR18112521 under BioProject PRJNA488469.
